# Patient reported outcome measures assessing quality of life in patients with an intestinal stoma: A systematic review

**DOI:** 10.1111/codi.16202

**Published:** 2022-06-16

**Authors:** Christian Valdemar Skibsted, Bente Thoft Jensen, Therese Juul, Helle Ø. Kristensen

**Affiliations:** ^1^ Department of Surgery Aarhus University Hospital Aarhus Denmark; ^2^ Department of Urology Aarhus University Hospital Aarhus Denmark; ^3^ Danish Cancer Society Centre for Research on Survivorship and Late Adverse Effects after Cancer in the Pelvic Organs Aarhus Denmark

**Keywords:** patient reported outcome measures, quality of life, stoma, systematic review, validation

## Abstract

**Aim:**

Living with a stoma can greatly influence quality of life. The purpose of this systematic review was to identify all patient reported outcome measures (PROMs) assessing health related quality of life (HRQoL) or similar constructs related to an intestinal stoma and to evaluate their level of validation.

**Methods:**

The study was reported in line with PRISMA guidelines. The protocol was registered in PROSPERO prior to the study. Eligible studies were any study investigating psychometric properties of a stoma‐specific PROM. The databases MedLine, Embase, CINAHL and Cochrane Libraries were searched for eligible studies. Studies were screened on title and abstract, then full‐text for eligibility. Data extraction on the study populations, PROM characteristics, psychometric properties as well as quality assessment using the COSMIN Risk of Bias checklist was performed.

**Results:**

In total, 40 studies were included concerning the development and/or validation of 21 PROMs. For most PROMs, few psychometric properties were assessed. In general, quality of content validity was poor, quality of construct validity and reliability was good. Assessment of responsiveness was lacking.

**Conclusion:**

This systematic review offers an overview of existing PROMs measuring stoma‐related HRQoL and their psychometric properties. A large number of PROMs exist and their measures overlap considerably. The PROMs generally have a low level of validation, emphasizing the need for future studies to further validate existing PROMs, rather than developing new ones.

## INTRODUCTION

Intestinal stoma formation involves diversion of the bowel to the skin, where the gut contents are emptied into a bag. Surgery with the possibility of intestinal stoma formation plays a major role in several conditions including colorectal cancer, inflammatory bowel disease and diverticular disease [1]. In this paper, “stoma” refers to an intestinal stoma.

It is well established that having a stoma can negatively impact health‐related quality of life (HRQoL) [2, 3, 4, 5]. HRQoL can be defined as an individual's perception of their physical and mental health [6, 7]. Stoma related problems may limit physical and social activities and affect daily life, thus reducing HRQoL [8, 9, 10, 11, 12]. Both physical and psychological factors can impact HRQoL. Physical factors include constipation, parastomal bulging, pain, fatigue, skin problems, seepage and odour; psychological include self‐efficacy, sexual problems, depressive feelings, dissatisfaction with appearance, change in clothing, travel difficulties, and worries about noises [13, 14]. Generally, complications after stoma formation are common with a systematic review reporting a frequency of 21%–70% [15].

Health‐related quality of life is a subjective measure and can be assessed using patient reported outcome measures (PROMs): tools to capture the patients own perception of health or HRQoL [16]. In patients with a stoma, stoma‐specific PROMs are preferable to generic PROMs as stoma‐specific symptoms and problems impact HRQoL [14, 17].

Numerous stoma‐specific PROMs have been developed and validated [3, 14, 17, 18]. While not every stoma‐specific PROM measures HRQoL directly, they all measure closely related constructs: adjustment to stoma, self‐efficacy, stoma acceptance, or stoma function. Stoma function, stoma acceptance, and self‐efficacy are closely associated to HRQoL [14, 19, 20] and adjustment to stoma defined as psychological, social and sexual functioning overlap considerably with HRQoL [21]. The existing stoma‐specific PROMs, however, differ in content, target population, and the extent of validation.

The validation of a PROM includes investigating and reporting on psychometric properties [7]. There are three main psychometric properties to address in such studies: reliability, validity and responsiveness. Reliability concerns how consistent an instrument is in its measurements, validity concerns whether a PROM measures what it intends and responsiveness concerns whether an instrument is able to detect a true change [22].

A targeted and validated PROM may assist researchers in obtaining relevant and valid data and assist clinicians in identifying patients with stoma‐related problems that potentially can be solved with improved HRQoL as a result [7].

We conducted a systematic review of the literature to identify and evaluate all available validated PROMs for assessment of HRQoL or related constructs and assess the body of evidence regarding the psychometric properties of each PROM.

## METHODS

### Study design

This review was performed in agreement with the recommendations of the “Preferred Reporting Items for Systematic Reviews and Meta‐Analyses” (PRISMA) [23, 24] and the protocol was registered in PROSPERO (CRD42020204905). Terminology of properties of instruments follows the COSMIN taxonomy where possible [25].

Studies were eligible for the review if they reported original data describing the development and/or assessment of psychometric properties of a PROM concerning stoma function, quality of life, HRQoL, overall satisfaction with life or well‐being in adults with a temporary or permanent intestinal stoma formed for any condition. Original articles in Danish, English, Norwegian or Swedish published in peer reviewed journals in any year were included. Exclusion criteria were studies investigating non‐stoma specific PROMs or PROMs not including a multi‐item stoma (sub)scale. Studies merely reporting translation or adaptation of an instrument were excluded.

### Search strategy and study selection

**FIGURE 1:**
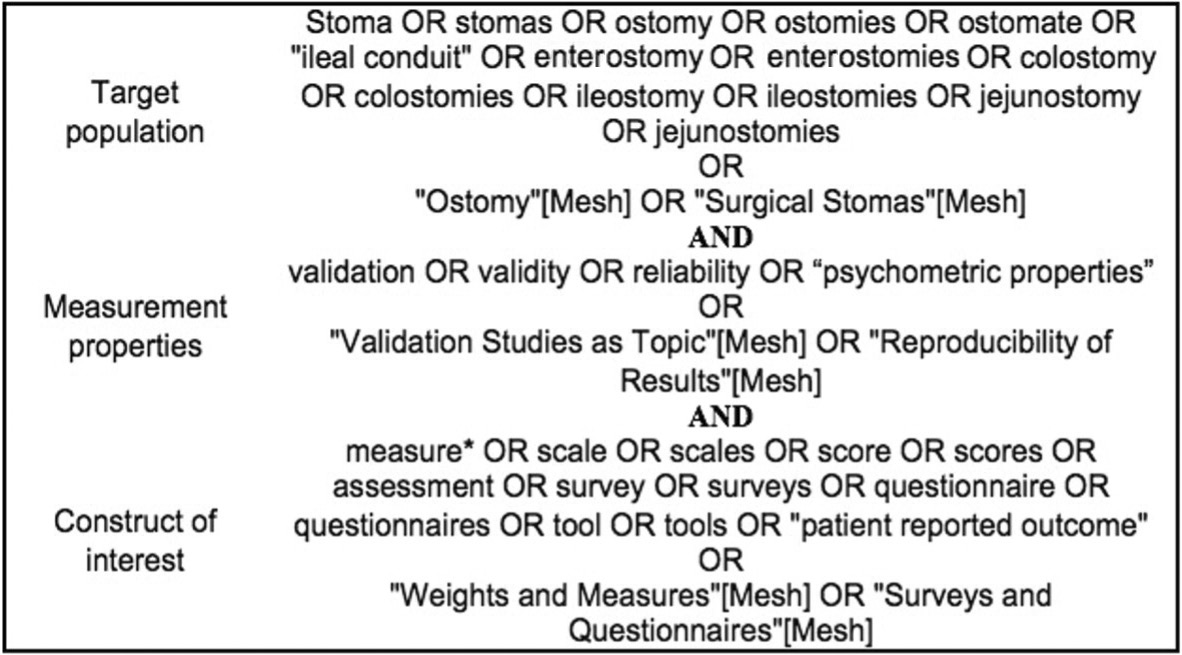
Search strategy for MedLine

A search strategy was developed for Medline, EMBASE, CINAHL and Cochrane Library with support from a librarian with experience in systematic reviews. Figure 1 shows the search strategy for MedLine. The strategy was adapted for each database (see Data S1).

All publications were uploaded to Covidence for screening. Covidence is an online tool developed for systematic reviews used for screening, data extraction and quality assessment [26]. First, duplicates were removed. Then, studies were screened for eligibility based on their title and abstract by two independent reviewers (CS and BT). Next, articles were assessed for eligibility based on full‐text by two independent reviewers (CS and TJ). In both screening rounds, conflicts were solved by consensus, or by a third party (HK) if consensus could not be reached. Lastly, the reference list of the included studies were hand searched for further relevant studies.

### Quality assessment and data synthesis

Two independent extractors (BT and HK) performed data extraction and assessed methodological quality of the included studies.

For each included study, characteristics of the study population, details regarding the PROM and psychometric properties were extracted. For the study population, this comprised: Number of included patients, type of stoma, disease characteristics, age and gender. For the PROM, this comprised: Target construct, target population, available languages and domains. For psychometric properties this comprised reported measures of: Content validity, construct validity, reliability and responsiveness.

The two reviewers assessed the methodological quality of all studies, where each psychometric property assessment was given a rating of inadequate (IA), doubtful (D), adequate (A), or very good (VG), using the COSMIN Risk of Bias Checklist [27, 28, 29]. To determine the rating, the corresponding COSMIN Risk of Bias box was completed, with the lowest rating in the box deciding the overall rating in concordance with the user manual [30].

Data was too heterogenous across studies for meta‐analysis. A narrative synthesis of the body of evidence concerning each PROM is presented with multiple studies concerning the same PROM addressed together. Characteristics and level of validation for each PROM are discussed.

## RESULTS

### Search strategy and study selection

**FIGURE 2:**
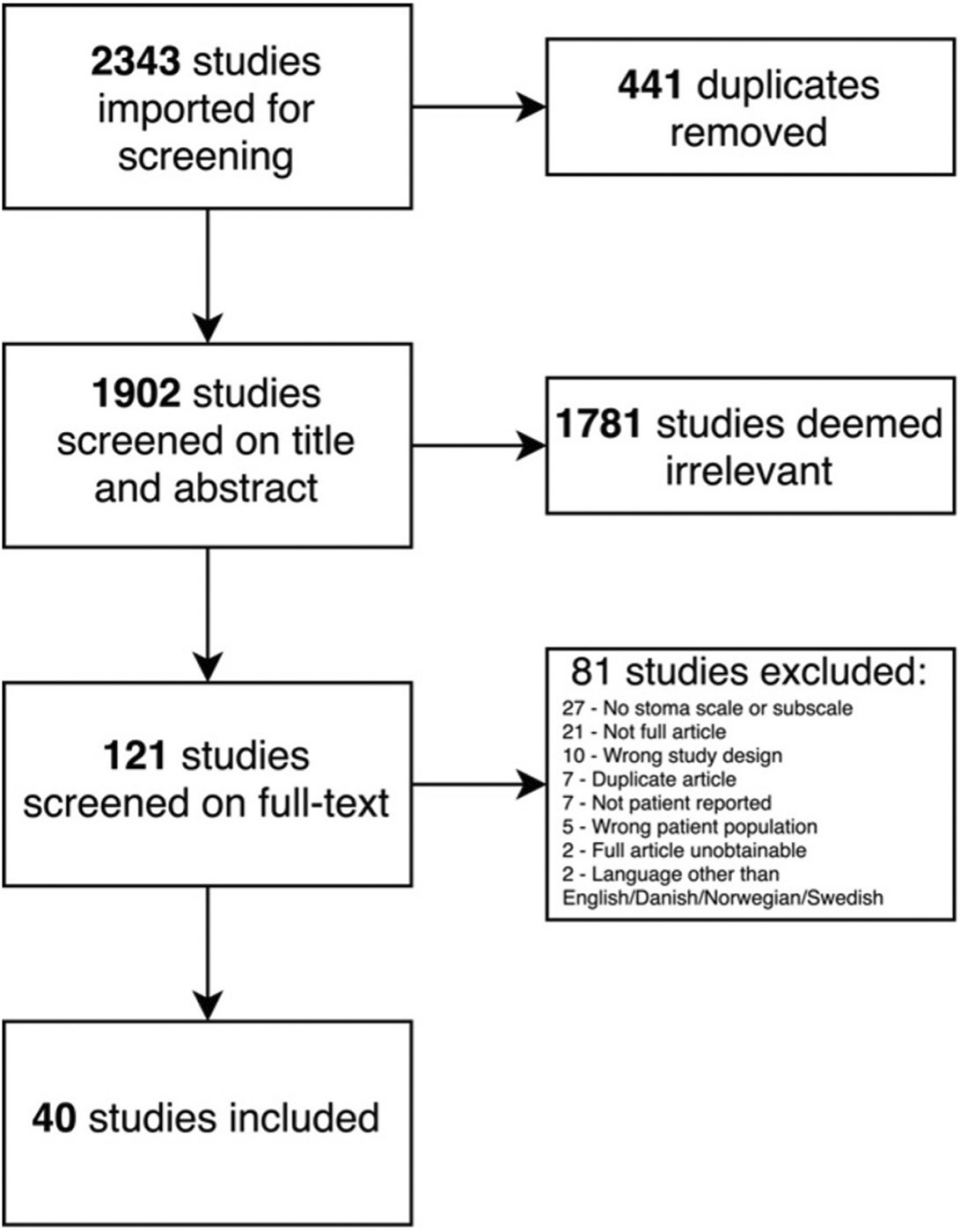
Study selection flowchart

The literature search was performed on 18 May 2021 and found 2343 studies. The screening process is presented in Figure 2. A total of 40 studies were included in the review. In both screening rounds, all conflicts were solved by consensus. Thorough hand‐search of the included studies' references yielded no additional studies.

### Quality assessment and data‐synthesis

Our study included 40 studies with data on 21 different PROMs (Table 1). An overview of PROMs and their abbreviations can be found in Table S1. The first study was published in 1983 while 34 studies were published during the past 20 years. The majority of study populations included more than one type of stoma and several underlying conditions. A total of 14 studies did not report on the disease characteristics of their study population and one study did not test its PROM in patients [31]. The mean age of the study populations was similar across studies, with mean age generally >50. Gender distribution varied across studies. Thirty‐seven studies were carried out in a single country, while three studies investigated an international study population. The size of populations ranged from 26–2470, with most populations consisting of 100–500 patients.

**TABLE 1:** Study characteristics

First author	Date of publication	Stoma‐PROM	Country	Number of patients	Age^a^	Gender distribution	Underlying disease	Type of stoma
Anaraki et al.	2014	COH‐QOL‐OQ	Iran	103	53.5 years (12.28)	56.3% male	N/A	Colostomy, ileostomy and urostomy
Bagnasco et al.	2017	SAQ	Italy	104	N/A	N/A	N/A	N/A
Baxter et al.	2006	SQOLS	US	100	59 years (15)	49% male	Cancer and IBD	Colostomy and ileostomy
Bekkers et al.	1996	SSES	Netherlands	59	44 years (17.3)	52.5% male	Cancer and IBD	Colostomy and ileostomy
Brydolf et al.	1994	OAS	Sweden	48	41.5 years (18–78)	37.5% male	N/A	Urostomy and ileostomy
Burckhardt	1990	OAS	US	164	58 years (N/A)	“About equally distributed”	N/A	N/A
Canova et al.	2013	Stoma‐QOL	Italy	251	62.5 years (15.3)	58.4% male	CRC, complications, IBD, other	Colostomy, ileostomy, multiple stomas
Colquhoun et al.	2006	CCFOFI	US	99	72 years (25–90)	59% male	N/A	Colostomy, ileostomy
Dellafiore et al.	2019	OAI‐23	Italy	230	71.62 years (12.3)	61.6% male	N/A	Colostomy, ileostomy
Gao et al.	2013	COH‐QOL‐OQ	China	370	67.3% over 60 years	64.05% male	N/A	Intestinal stoma, cystostomy
Grant et al.	2004	COH‐QOL‐OQ	US	1513	69.5 years (12.77)	47% male	Cancer and non‐cancer	Colostomy, ileostomy, urostomy, multiple ostomies
Harisi et al.	2004	CRC_QOL	Hungary	132	65 years (33–82)	N/A	Colorectal cancer	Colostomy
Indrebø et al.	2014	OAS	Norway	158	64 years (29–91)	56% male	Cancer, IBD, other	Colostomy, ileostomy, urostomy
Indrebø et al.	2021	OAS	Norway	302	63.3 yr (14.9)	56.6% male	Cancer and IBD	Colostomy, ileostomy, urostomy, two stomas
Jin et al.	2020	CDS	China	423	61.31 years (7.48)	58.1% male	Colorectal cancer	Colostomy
Karaçay et al.	2020	SSES	Turkey	174	54 years (14.53)	59.8% male	Cancer, other	Colostomy, ileostomy
Kluka et al.	1996	OCS	Canada	20 patients/20 partners	85% under 65 years	75% male	N/A	Colostomy, urostomy
Konjevoda et al.	2020	COH‐QOL‐OQ	Croatia	302	59.3 years (15.8)	60.3% male	Cancer, IBD, diverticulitis, ileus, injury	Colostomy, ileostomy, urostomy
Kristensen et al.	2020	CIS	Denmark	252	69 years (21–95)	41% male	Benign conditions: diverticulitis, incontinence, fistula, Crohns, obstipation, ileus, anastomotic leakage, other	Colostomy
Kristensen et al.	2021	CIS	Australia, China, Denmark, Netherlands, Portugal, Spain, Sweden	2470	74.4 years (13–96)	63% male	Colorectal cancer	Colostomy
Krouse et al.	2006	COH‐QOL‐OQ	US	239 ostomates / 272 controls	68.8 years (12.4)	92% male	CRC, IBD, acute inflammation, benign tumour, other	Colostomy, ileostomy
Lai et al.	2018	Stoma‐QOL	Canada	120	58 years (19–86)	62.5% male	Non‐cancer	Temporary ileostomies and colostomies
Lim et al.	2017	ACHC (Stoma)	Singapore	100	64 years (9.7)	59% male	Colorectal cancer	Colostomy, ileostomy, double barrel stoma
Mayadevi et al.	2019	COH‐QOL‐OQ	India	92	54.9 years (9.9)	58.7% male	Cancer	Colostomy, ileostomy, urostomy
Mohler et al.	2008	COH‐QOL‐OQ	US	284	72.4 years (10.3)	58.7% male	Colorectal cancer	Intestinal stoma
Nafees et al.	2017	Ostomy‐Q	UK	200	42.5% over 60 years	49% male	N/A	Ileostomy, colostomy
Nafees et al.	2018	OLIT	UK, US, France, Denmark	340	59.4 years (13.2)	45.6% male	N/A	Colostomy, Ileostomy
Nordström et al.	1995	ADM	Sweden	26	69 years (49–85)	46.2% male	N/A	Continent and conventional stoma
Olbrisch	1983	OAS	US	53	(19–83 years)	54.7% male	N/A	Colostomy, ileostomy, urostomy
Oliveira et al.	2017	Stoma‐QOL	Brazil	111	84.7% over 50 years	51.4% male	Cancer and non‐cancer	Permanent and temporary colostomy and ileostomy
Prieto et al.	2005	Stoma‐QOL	Denmark, France, Spain, Germany	182	53.4 years (15.3)	52.5% male	Crohn's, cancer, diverticulitis, other	Colostomy, ileostomy
Santos et al.	2020	OAI‐23	Brazil	191	58.9 years (14.7)	42.9% male	Cancer and non‐cancer	Colostomy, ileostomy and urostomy
Santos et al.	2021	COH‐QOL‐OQ	Brazil	215	59 years (16.7)	51.6% male	Colorectal cancer, other	Permanent and temporary colostomy, ileostomy and urostomy
Simmons et al.	2009	OAI‐23	UK	570	67.1 years (17–91)	51% male	N/A	Colostomy, ileostomy, urostomy, two stomas
Sodhi et al.	2012	PSAT	India	0	Not studied in patients	Not studied in patients	Not studied in patients	
Sousa et al.	2015	EAOE	Portugal	256	62.4 years (13.13)	52% male	N/A	Intestinal stoma, urostomy
Thyø et al.	2016	CIS	Denmark	610	68 years (59–76)	65% male	Colorectal cancer	Permanent colostomy
Villa et al.	2019	OSCI + CC‐OSCI	Italy	468 patients/227 caregivers	68 years (N/A)	“Mostly male” for patients / 78.41% female for caregivers	Cancer, other	Colostomy, ileostomy, urostomy
Wendel et al.	2014	BFI	US	183	74.7 years (11.1)	65.6% male	Cancer	Colostomy, ileostomy
Zhang et al.	2015	OAS	China	207	56.7 years (12.3)	72% male	Colorectal cancer	Permanent colostomy

Abbreviations: ACHC (Stoma), Acceptance of Chronic Health Conditions Stoma Scale; ADM, Acceptance of Disability Scale Modified; BFI, Bowel Function Index (modified); CC‐OSCI, Caregiver Contribution to Self‐Care in Ostomy Patient Index; CCFOFI, Cleveland Clinic Florida Ostomy Function Index; CDS, Colostomy Disgust Scale; CIS, Colostomy Impact Score; COH‐QOL‐OQ, City of Hope Quality of Life Ostomy Questionnaire; CRC_QOL, Colorectal cancer specific questionnaire on quality of life; EAOE, Elimination Ostomy Adjustment Scale; FBCS, Fragebogens zu Belastungssituationen bei Colostoma (Colostomy Burden Questionnaire); N/A, not available; OAI‐23, Ostomy Adjustment Inventory‐23; OAS, Ostomy Adjustment Scale; OCS, Ostomy Concerns Scale; OLIT, Ostomy Leak Impact Tool; OSCI, Ostomy Self‐Care Index; PSAT, Peristomal Skin Assessment Tool; SAQ, Stoma Acceptance Questionnaire; SQOLS, Stoma Quality Of Life Scale; SSES, Stoma Self‐Efficacy Scale; Stoma‐QOL, Stoma Quality of Life Questionnaire.

^a^ Age reported as mean (SD), as median (range–range), or as other way of reporting.

The psychometric properties investigated in each study are presented in Table 2, along with a quality assessment of each psychometric property reported. A total of 38 studies investigated more than one psychometric property. In total, 15 studies reported on the development of a PROM, and 16 assessed content validity. Structural validity was investigated in 22 studies, 35 assessed internal consistency and 28 assessed hypothesis testing for construct validity. Reliability was reported in 20 studies. A single study assessed responsiveness [32]. No studies assessed cross‐cultural validity, measurement error or criterion validity.

**TABLE 2:** Assessed psychometric properties and quality assessment

Studies: First author, date of publication	Stoma‐PROM	Development	Content validity	Structural validity	Internal consistency	Cross‐cultural validity	Reliability	Measurement error	Criterion validity	Hypothesis‐testing for construct validity	Responsiveness
Anaraki et al., 2014 [33]	COH‐QOL‐OQ	‐	D	‐	VG	‐	‐	‐	‐	A	‐
Bagnasco et al., 2017 [20]	SAQ	D	‐	IA	VG	‐	‐	‐	‐	‐	‐
Baxter et al., 2006 [17]	SQOLS	IA	‐	‐	VG	‐	IA	‐	‐	VG	‐
Bekkers et al., 1996 [19]	SSES	IA	‐	IA	VG	‐	‐	‐	‐	A	‐
Brydolf et al., 1994 [34]	OAS	‐	‐	‐	IA	‐	D	‐	‐	A	‐
Burckhardt, 1990 [35]	OAS	‐	‐	A	D	‐	A	‐	‐	VG	‐
Canova et al., 2013 [36]	Stoma‐QOL	‐	‐	VG	VG	‐	‐	‐	‐	‐	‐
Colquhoun et al., 2006 [37]	CCFOFI	‐	‐	‐	‐	‐	‐	‐	‐	VG	‐
Dellafiore et al., 2019 [38]	OAI‐23	‐	D	A	VG	‐	‐	‐	‐	VG	‐
Gao et al., 2013 [39]	COH‐QOL‐OQ	‐	D	VG	VG	‐	D	‐	‐	‐	‐
Grant et al, 2004 [3]	COH‐QOL‐OQ	‐	A	A	VG	‐	‐	‐	‐	VG	‐
Harisi et al., 2004 [40]	CRC_QOL	IA	‐	‐	VG	‐	D	‐	‐	A	‐
Indrebø et al, 2014 [41]	OAS	‐	D	‐	VG	‐	D	‐	‐	VG	‐
Indrebø et al, 2021 [42]	OAS	‐	D	VG	D	‐	‐	‐	‐	‐	‐
Jin et al., 2020 [43]	CDS	IA	D	VG	VG	‐	‐	‐	‐	‐	‐
Karaçay et al., 2020 [44]	SSES	‐	D	VG	VG	‐	A	‐	‐	‐	‐
Kluka et al., 1996 [45]	OCS	D	‐	‐	VG	‐	D	‐	‐	‐	‐
Konjevoda et al., 2020 [46]	COH‐QOL‐OQ	‐	‐	VG	VG	‐	VG	‐	‐	‐	‐
Kristensen et al, 2020 [47]	CIS	‐	‐	‐	‐	‐	‐	‐	‐	VG	‐
Kristensen et al., 2021 [48]	CIS	‐	‐	‐	‐	‐	A	‐	‐	VG	‐
Krouse et al., 2006 [49]	COH‐QOL‐OQ	‐	‐	‐	VG	‐	‐	‐	‐	‐	‐
Lai et al., 2018 [50]	Stoma‐QOL	‐	‐	IA	D	‐	‐	‐	‐	A	‐
Lim et al., 2017 [51]	ACHC (Stoma)	‐	D	‐	VG	‐	A	‐	‐	VG	‐
Mayadevi et al., 2019 [52]	COH‐QOL‐OQ	‐	D	‐	VG	‐	IA	‐	‐	‐	‐
Mohler et al., 2008 [53]	COH‐QOL‐OQ	‐	‐	‐	VG	‐	‐	‐	‐	VG	‐
Nafees et al, 2017 [32]	Ostomy‐Q	IA	D	A	VG	‐	A	‐	‐	VG	A
Nafees et al., 2018 [54]	OLIT	A	A	‐	D	‐	A	‐	‐	VG	‐
Nordström et al., 1995 [55]	ADM	‐	‐	‐	VG	‐	‐	‐	‐	VG	‐
Olbrisch, 1983 [21]	OAS	IA	‐	IA	D	‐	D	‐	‐	A	‐
Oliveira et al., 2017 [56]	Stoma‐QOL	‐	‐	‐	IA	‐	IA	‐	‐	A	‐
Prieto et al., 2005 [18]	Stoma‐QOL	IA	‐	A	D	‐	A	‐	‐	‐	‐
Santos et al., 2020 [57]	OAI‐23	‐	‐	D	VG	‐	D	‐	‐	VG	‐
Santos et al., 2021 [58]	COH‐QOL‐QQ	‐	‐	VG	IA	‐	‐	‐	‐	VG	‐
Simmons et al, 2009 [59]	OAI‐23	D	‐	D	VG	‐	D	‐	‐	VG	‐
Sodhi et al., 2012 [31]	PSAT	IA	D	‐	‐	‐	‐	‐	‐	‐	‐
Sousa et al., 2015 [60]	EAOE	D	D	A	VG	‐	‐	‐	‐	VG	‐
Thyø et al., 2016 [14]	CIS	IA	‐	‐	‐	‐	‐	‐	‐	VG	‐
Villa et al., 2019 [61]	OSCI + CC‐OSCI	IA	D	A	VG	‐	‐	‐	‐	VG	‐
Wendel et al., 2014 [62]	BFI	‐	‐	A	VG	‐	‐	‐	‐	VG	‐
Zhang et al., 2015 [63]	OAS	‐	D	A	VG	‐	A	‐	‐	VG	‐

*Not*e: VG, very good; A, adequate; D, doubtful; IA, inadequate; −, not assessed.

Abbreviations: ACHC (Stoma), Acceptance of Chronic Health Conditions Stoma Scale; ADM, Acceptance of Disability Scale Modified; BFI, Bowel Function Index (modified); CC‐OSCI, Caregiver Contribution to Self‐Care in Ostomy Patient Index; CCFOFI, Cleveland Clinic Florida Ostomy Function Index; CDS, Colostomy Disgust Scale; CIS, Colostomy Impact Score; COH‐QOL‐OQ, City of Hope Quality of Life Ostomy Questionnaire; CRC_QOL, Colorectal cancer specific questionnaire on quality of life; EAOE, Elimination Ostomy Adjustment Scale; FBCS, Fragebogens zu Belastungssituationen bei Colostoma (Colostomy Burden Questionnaire); OAI‐23, Ostomy Adjustment Inventory‐23; OAS, Ostomy Adjustment Scale; OCS: Ostomy Concerns Scale; OLIT, Ostomy Leak Impact Tool; OSCI, Ostomy Self‐Care Index; PSAT, Peristomal Skin Assessment Tool; SAQ, Stoma Acceptance Questionnaire; SQOLS, Stoma Quality Of Life Scale; SSES, Stoma Self‐Efficacy Scale; Stoma‐QOL, Stoma Quality of Life Questionnaire.

The quality of development and content validation studies were generally low, with only three ratings of “adequate” (A) and no ratings of “very good” (VG) in 31 assessments. Generally, the validation of PROMS for internal consistency, structural validity and hypothesis testing for construct validity had high quality with a majority ranking VG or A. For reliability, 11 out of 20 studies were rated “inadequate” (IA) or “doubtful” (D). The single assessment of responsiveness was ranked as A.

Table S2 displays data for each of the PROMs developed or validated in the included studies, which includes a summary of the broadest population in which the PROM has been studied, number of items, domains/subscales, available languages and in which studies the PROM has been validated. Table S3 displays study results including reported data on content validity, construct validity, reliability and responsiveness. In both tables, the PROMs are grouped based on which construct they aim to measure: Adjustment to stoma, stoma related HRQoL, self‐efficacy, stoma acceptance or stoma‐function.

#### Adjustment to stoma

Five PROMs aim to assess adjustment to stoma [21, 45, 55, 59, 60]. The Ostomy Adjustment Scale (OAS) and Ostomy Adjustment Index‐23 (OAI‐23) have both been validated in more than one study. Both cover domains concerning social interaction and psychological status in relation to adjustment. Development or content validity assessment is performed in several studies but with quality ranked as D or IA. The quality of construct validity were A or VG for all PROMs measuring adjustment to stoma. The reliability for the OAS was assessed by test–retest in five studies with intraclass correlation coefficients ranging from 0.59–0.90.

#### Health‐related quality of life in stoma patients

Five PROMs aim to assess HRQoL in stoma patients [3, 17, 32, 40]. They generally contain domains regarding physical, psychological and social state. Stoma‐QOL has been validated in four studies and COH‐QOL‐OQ in eight studies, they are validated for several stoma types and underlying conditions, and they are available in many languages. The structural validity, internal consistency, and construct validity of COH‐QOL‐OQ were generally assessed with a rating of A or VG. Generally, the quality of assessments was better for COH‐QOL‐OQ than for Stoma‐QOL. Four of the eight studies concerning COH‐QOL‐OQ used factor analysis to assess construct validity. The Ostomy‐Q was the only PROM for which responsiveness was assessed with a ranking of A.

#### Self‐efficacy in stoma patients

Two PROMs measure self‐efficacy in stoma patients and one in caregivers [19, 61]. They have been tested in populations of different stoma types and conditions. Their construct validity and reliability have been assessed with a ranking generally A or VG. The three studies all report strong internal consistency with Cronbachs alpha across all subscales of the three studies ranging from 0.912–0.972.

#### Stoma acceptance

Three PROMs assess stoma acceptance [20, 43, 51]. All are validated in one study each. All three have had their internal consistency assessed with a ranking of VG and reported Cronbachs alpha ranging 0.85–0.94.

#### Stoma function

Five PROMs measure stoma function [14, 31, 37, 54, 62]. CIS is validated in three studies, the four others in one study each. CIS have been validated for colostomy patients with any condition, while CCFOFI, BFI and OLIT have been validated for colostomy and ileostomy patients. PSAT were not validated in patients. OLIT had its content validity, construct validity and reliability assessed with a quality ranking of A or VG. The six studies including patients all showed convergent validity with significant correlation between the studied scales and generic PROMs measuring HRQoL.

## DISCUSSION

This systematic review summarized and critically evaluated published evidence on stoma‐specific PROMs and their psychometric properties. Our intention was to provide an overview of available tools, and to support selection of the best suited PROM for a specific clinical or research purpose.

This review identified 21 PROMs, where only one had both content validity, construct validity, reliability and responsiveness assessed [32]. The differences between types of PROMs and differences in the assessment of their psychometric properties hinders direct comparisons between studies. Furthermore, the lack of a standardized use of taxonomy and validation procedure poses a problem, and many PROMs are validated only for few psychometric properties and/or in limited patient groups. Fewer PROMs with less overlap in construct and with more complete assessment of psychometric properties would increase applicability of said PROMs and would improve the basis for health professionals to choose the right PROM for their purpose [7].

The quality of assessments was generally good for structural validity, internal consistency, reliability, and construct validity; and generally poor for development and content validity. A reason may be that development and assessment of content validity is more demanding and time consuming and may be difficult to describe, which is also reflected in the COSMIN risk of bias checklist being more extensive for development and content validation [28, 29, 30]. The steps are important to follow, however, as correct inclusion of both experts and patients is required for PROMs to focus on relevant constructs [64].

No studies investigated the cross‐cultural validity, measurement error or criterion validity of PROMs. Cross‐cultural validity requires comparison of the validity between two patient groups, where cultural differences are expected to impact the construct [7]. As religious beliefs and body image are associated with stoma‐related HRQoL [17, 65] and vary across cultures, cross‐cultural validation might reveal that PROMs need to weight items regarding these factors differently for different cultures. Measurement error parameters are related to reliability parameters, are often not reported, but give valuable information on the precision of PROMs [7]. Criterion validity requires a gold‐standard measurement to compare PROMs with. Since a gold‐standard does not exist for measuring stoma‐related HRQoL, it cannot be assessed.

Responsiveness was formally evaluated in only one study [32]. Two recent intervention studies [66, 67] used the SSES and Stoma‐QOL to measure change in self‐efficacy and HRQoL, but the responsiveness of these PROMs remains unassessed. In both studies, the instruments found a change where change was expected, which suggests responsiveness. However, if an intervention study unknowingly uses an unresponsive PROM, they will falsely conclude that an intervention is without effect, even if there is one, since the PROM would not be able to detect it [7]. This emphasizes the importance of further validation of existing PROMs, to ensure that they produce valid, useful and adequate data for future studies.

## STRENGTHS AND LIMITATIONS

A primary strength of this study is its meticulousness. We have searched several large, relevant databases of scientific, medical studies. We have used a strong search strategy developed in cooperation with a librarian with experience in systematic reviews. Another strength is that every step of the screening and data extraction was performed by two researchers independently, thus reducing the risk of error or bias in screening, data extraction and quality assessment.

This study has some limitations. We limited our search and inclusion criteria to English, Danish, Norwegian and Swedish, but we excluded only one article because of language. Another limitation is the exclusion of studies, for which only abstracts or poster presentations have been published, where relevant data may exist but is unavailable.

In this systematic review, we strictly adhered to the comprehensive guidance laid out by the COSMIN initiative [27]. When assessing the quality of the studies, the COSMIN Risk of Bias checklist dictates a “worst score counts” principle. This is arguably overly strict, and should be considered as a limitation to the COSMIN method. The COSMIN taxonomy was introduced in 2010 and thus relatively new [25]. Adhering to it might be a limitation of our literature search, if older relevant studies using a different taxonomy were not identified. However, no additional studies were found through hand search, proving the search strategy to be strong.

Another limitation of using COSMIN taxonomy is that 14 of the 40 included studies precede it. Several instruments were created and validated in a time without clear international standards. Therefore, they often lack steps or reporting of steps in development and validation that are currently expected. As a result, COSMIN ratings should be interpreted with caution, as lower ratings do not necessarily reflect a lack of quality in a study, but rather reveal a lack of reporting standards.

Our systematic review only included PROMs with a multi‐item stoma scale or subscale. The European Organization for Research and Treatment of Cancer (EORTC) Quality of Life Questionnaire for Colorectal Patients (EORTC QLQ‐CR29) is a 29‐item tool frequently used to measure HRQoL in colorectal cancer patients with or without a stoma [68, 69]. Although this tool includes a number of single items related to stoma function, it does not contain a multi‐item stoma subscale and therefore it was excluded from this review.

## CONCLUSION

This systematic review provides an overview of available stoma‐specific PROMs. We have included 40 studies reporting the development and/or psychometric properties of a total of 21 PROMs. Hopefully, this overview can guide researchers and clinicians in selection of a PROM best suited for their intended purpose. The large number of PROMs covers the different aspects of stoma function and stoma related HRQoL extensively and overlap considerably. Combined with a generally low level of validation, the results of this review emphasizes a strong need for future studies to further validate existing PROMs, rather than developing new ones.

## CONFLICT OF INTEREST

None.

## FUNDING INFORMATION

No funding to declare.

## AUTHOR CONTRIBUTIONS

Christian Valdemar Skibsted screened studies for eligibility and drafted the publication. Helle Ø. Kristensen performed data extraction and critically revised the manuscript. Bente Thoft Jensen screened studies for eligibility, performed data extraction, and provided important feedback to the manuscript. Therese Juul screened studies for eligibility and provided important feedback to the manuscript.

## Supporting information


Appendix S1

Tables S1‐S3

Data S1

## Data Availability

Data sharing is not applicable to this article as no new data were created or analyzed in this study.
