# Supplementary material for: Patient reported outcome measures assessing quality of life in patients with an intestinal stoma: A systematic review
Source: Colorectal Dis. 2022 Jun 16;24(10):1128–39. doi: 10.1111/codi.16202 (PMC9796962; doi:10.1111/codi.16202)
Supplement: Supplementary file 2 — Tables S1‐S3 [file CODI-24-1128-s003.docx]

# Supplementary tables

**Supplementary table 1 - Overview and abbreviations of patient reported outcome measures (PROMs)**

| **Abbreviation** | **Name of PROM** | **Year of first study** | **Number of studies** |
| --- | --- | --- | --- |
| ACHC (Stoma) | Acceptance of Chronic Health Conditions Stoma Scale | 2017 | 1 |
| ADM | Acceptance of Disability Scale Modified | 1995 | 1 |
| BFI | Bowel Function Index (modified) | 2014 | 1 |
| CC-OSCI | Caregiver Contribution to Self-Care in Ostomy Patient Index | 2019 | 1 |
| CCFOFI | Cleveland Clinic Florida Ostomy Function Index | 2006 | 1 |
| CDS | Colostomy Disgust Scale | 2020 | 1 |
| CIS | Colostomy Impact Score | 2016 | 3 |
| COH-QOL-OQ | City of Hope Quality of Life Ostomy Questionnaire | 2004 | 8 |
| CRC_QOL | Colorectal cancer specific questionnaire on quality of life | 2004 | 1 |
| EAOE | Elimination Ostomy Adjustment Scale | 2015 | 1 |
| OAI-23 | Ostomy Adjustment Inventory-23 | 2009 | 3 |
| OAS | Ostomy Adjustment Scale | 1983 | 6 |
| OCS | Ostomy Concerns Scale | 1996 | 1 |
| OLIT | Ostomy Leak Impact Tool | 2018 | 1 |
| OSCI | Ostomy Self-Efficacy Index | 2019 | 1 |
| Ostomy-Q | Ostomy-Q | 2017 | 1 |
| PSAT | Peristomal Skin Assessment Tool | 2012 | 1 |
| SAQ | Stoma Acceptance Questionnaire | 2017 | 1 |
| SQOLS | Stoma Quality Of Life Scale | 2006 | 1 |
| SSES | Stoma Self-Efficacy Scale | 1996 | 2 |
| Stoma-QOL | Stoma Quality of Life Questionnaire | 2005 | 4 |

**Supplementary table 2 - Characteristics of patient reported outcome measures (PROMs)**

| **Construct measured** | **Instrument** | **Available languages** | **Broadest study population (Type of stoma,**  **underlying conditions)** | **Number of items** | **Domains/ subscales** | **Studies (1st author, publ. year)** |
| --- | --- | --- | --- | --- | --- | --- |
| Adjustment to stoma | OAS | Swedish English Norwegian Danish Chinese | Colostomy, ileostomy, urostomy. Cancer, IBD, other. | 34 | 3 Physical function, psycholocical state, social interaction  “New subscales”: 7  Daily activities, knowledge and skills, self-esteem, psychological/existential, health, health professionals and sexuality. | Brydolf et al, 1994  Burckhardt, 1990 Indrebø et al, 2014  Indrebø et al, 2021 Olbrisch, 1983  Zhang et al, 2015 |
|  | OAI-23 | Italian English Chinese Japanese Nepali Norwegian  Portugese | Colostomy, ileostomy, urostomy, two stomas. N/A. | 23 | 4 Acceptance, anxious preoccupation, social engagement, anger. | Dellafiore et al, 2019  Santos et al, 2020  Simmons et al, 2009 |
|  | OCS | English | Colostomy, urostomy. N/A. | 48 | 1 | Kluka et al, 1996 |
|  | EAOE | Portugese | Intestinal stoma, urostomy N/A. | 39 | 6 Self-concept, self-care, acceptance, hope, sexual interaction, social interaction | Sousa et al, 2015 |
|  | ADM | English  Swedish | Continent and coventional stoma. N/A. | 50 | 4 Enlargement of scope of values, subordination of physique, containment of disability effects, transformation from comparative values to asset values | Nordström et al, 1995 |
| HRQoL in stoma patients | SQOLS | English | Colostomies and ileostomies. Benign and malignant disease. | 21 | 5 Work/social functioning, sexual/body image, stoma function, financial concerns, skin irritation | Baxter et al, 2006 |
|  | COH-QOL-Ostomy Questionnaire | Persian English Chinese Portugese  Brazilian-Portugese Turkish Croatian Malayalam | Colostomy, ileostomy, urostomy, multiple stomas. Cancer, IBD, diverticulitis, ileus, injury. | 43 | 4 Physical well-being, psychological well-being, social well-being, spiritual well-being | Anaraki et al, 2014  Gao et al, 2013  Grant et al, 2004  Konjevoda et al, 2020  Krouse et al, 2006  Mayadevi et al, 2019  Mohler et al, 2008  Santos et al, 2021 |
|  | CRC_QOL | Hungarian English | Colostomy. Colorectal cancer. | 62 | 4 Physical status, functional status, emotional status, social status | Harisi et al, 2004 |
|  | Stoma-QOL | English Danish German Swedish Spanish French Portugese | Colostomy, ileostomy. Cancer, IBD, complications, other. | 20 | 4 Sleep, sexual activity, relationships to family and close friends, social relations to other than family and close friends | Lai et al, 2018  Oliveira et al, 2017  Prieto et al, 2005  Canova et al, 2013 |
|  | The Ostomy-Q | English | Ileostomy, colostomy. N/A. | 23 | 4 Discreetness, comfort, confidence, social Life | Nafees et al, 2017 |
| Self-efficacy in stoma patients | SSES | Turkish English | Colostomy, ileostomy. Cancer and IBD. | 22 | 2 Stoma care self-efficacy, social self-efficacy | Bekkers et al, 1996  Karaçay et al, 2020 |
|  | CC-OSCI | Italian English | Colostomy, ileostomy, urostomy. Cancer, other. | 22 | 3 Contribution to maintenance, contribution to monitoring, contribution to management | Villa et al, 2019 |
|  | OSCI | Italian English | Colostomy, ileostomy, urostomy. Cancer, other. | 32 | 4 Maintenance, monitoring, management, confidence | Villa et al, 2019 |
| Stoma  acceptance | ACHC (Stoma) | English Chinese | Colostomy, ileostomy, double barrel stoma. Colorectal cancer. | 10 | 1 | Lim et al, 2017 |
|  | SAQ | English Italian | N/A. N/A. | 17 | 3 Importance, trust, self-determination and responsibility | Bagnasco et al, 2017 |
|  | CDS | Chinese  English | Colostomy.  Colorectal cancer. | 22 | 2  Disgust related to stoma symptoms, disgust related to interpersonal interactions. | Jin et al, 2020 |
| Stoma function | CIS | Chinese  Danish  Dutch English  Portugese  Spanish  Swedish | Colostomy Cancer and benign conditions | 7 | 1 | Thyø et al, 2016  Kristensen et al, 2020  Kristensen et al, 2021 |
|  | CCFOFI | English | Colostomy, ileostomy. N/A. | 7 | 1 | Colquhoun et al, 2006 |
|  | BFI | English | Colostomy, ileostomy. Cancer. | 18 | 3 Frequency, dietary, soilage | Wendel et al, 2014 |
|  | PSAT | N/A | Not studied in patients | 25 | 1 | Sodhi et al, 2012 |
|  | OLIT | English French Danish | Colostomy, ileostomy. N/A. | 22 | 3 Emotional impact, usual and social activities, coping and control | Nafees et al, 2018 |
| ACHC (Stoma): Acceptance of Chronic Health Conditions Stoma Scale; ADM: Acceptance of Disability Scale Modified; BFI: Bowel Function Index (modified); CC-OSCI: Caregiver Contribution to Self-Care in Ostomy Patient Index; CCFOFI: Cleveland Clinic Florida Ostomy Function Index; CDS: Colostomy Disgust Scale; CIS: Colostomy Impact Score; COH-QOL-OQ: City of Hope Quality of Life Ostomy Questionnaire; CRC_QOL: Colorectal cancer specific questionnaire on quality of life; EAOE: Elimination Ostomy Adjustment Scale; OAI-23: Ostomy Adjustment Inventory-23; OAS: Ostomy Adjustment Scale; OCS: Ostomy Concerns Scale; OLIT: Ostomy Leak Impact Tool; OSCI: Ostomy Self-Care Index; PSAT: Peristomal Skin Assessment Tool; SAQ: Stoma Acceptance Questionnaire; SQOLS: Stoma Quality Of Life Scale; SSES: Stoma Self-Efficacy Scale; Stoma-QOL: Stoma Quality of Life Questionnaire. | | | | | | |

**Supplementary table 3 - Results from included studies**

| **Construct measured** | **Stoma-Prom** | **Studies: 1st author, publ. year** | **Content validity (Development and content validity)** | **Construct validity (Structural validity, cross-cultural validity and hypothesis testing for construct validity)** | **Reliability (Internal consistency and reliability)** | **Responsiveness** |
| --- | --- | --- | --- | --- | --- | --- |
| Adjustment to stoma | OAS | Brydolf et al, 1994 | - | Pearson correlation coefficient between OAS and a QOL Visual analouge scale: 0.67 | Cronbachs alpha: 0.95 Test-retest reliability coefficient: 0.59 | - |
|  |  | Burckhardt, 1990 | - | Exploratory factor analysis yielded 5 factors. OAS correaltion coefficient with 15-item QOL scale: 0.40 OAS correlation coefficient with 5-item depression index: -0.53 Known-groups validity: Disciminates between cancer and non-cancer patients. No correlation with time since surgery. | Cronbachs alpha: 0.90 Item to total scale correlation: 0.43 (range 0.13-0.73) Test-retest correlation: 0.66 | - |
|  |  | Indrebø et al, 2014 | Five WOC nurses found the OAS acceptable. | Pearson correlation coefficient between OAS and: - Quality of life scale 0.44 - Hospital anxiety and depression scale - Anxiety: -0.37 - Hospital anxiety and depression scale - Depression: -0.40 - SF36 scale scores: Range 0.28-0.45 - General Self-Efficacy scale: 0.32 | Cronbachs alpha: 0.93 Test-retest reliability (Pearson coefficient): 0.69 | - |
|  |  | Indrebø et al, 2021 | - | Confirmatory factor analysis: RMSEA = 0.053 (90% CI, 0.045–0.060), CFI = 0.913 and TLI = 0.904. The pairwise covariance between subscales ranged between 0.12 and 0.48. | Composite reliability values >0.7 for all subscales. | - |
|  |  | Olbrisch, 1983 | The OAS was developed based on a review of the literature. In addition, three ostomy patients and three professionals were asked to generate items. | Exploratory factor analysis yielded 5 factors. Convergent validity: No significant correlation between the OAS and Marlowe-Crowne Social Desirability Scale (r= - .14) or on the Texasa Social Behaviour Inventory (r = .02). Known-groups-validity: Discriminates between stoma patients and students simulating stoma patients. | Cronbachs alpha: 0.87 Test-retest correlation coefficient: 0.72 | - |
|  |  | Zhang et al, 2015 | The content validity, derived from the ratings of 5 members of an expert panel, showed a content validity index of 0.91 with a mean score for each item ranging from 3.0 to 4.0 | Exploratory factor analysis yielded 4 factors. Convergent validity: The chinese OAS score was significantly correlated with the Chinese version of SSES (r= 0.632, p<0.001) and Stoma Self-Care Scale General Version (r= 0.463, p<0.001) | Cronbachs alpha: 0.915, for each factor range 0.754-0.893. Split-half Spearman-Brown coefficient: 0.871. Test-retest reliability: ICC for a 2-week interval was 0.902 | - |
|  | OAI-23 | Dellafiore et al, 2019 | Content validity for all 23 items were considered relevant (Content Validity Ratio > 0.70) and appropriate (Item-level-Content Validity Index and Scale Level-Content Validity Index > 0.90) by a panel of 15 professionals. | Exploratory factor analysis yielded 3 factors. Known-groups-validity: Dicriminates between men and women, older and younger patients and high/low BMI. | Chronbachs alfa: 0.91, for each factor range 0.87-0.93 | - |
|  |  | Simmons et al, 2009 | The OAI-23 was developed based on a translation of Ostomates Self Adjustment Score reviewed by a reasearch group and pilot tested in a patient group. | Exploratory factor analysis yielded 4 factors. Convergent validity: The OAI-23 showed significant correlation to the Acceptance of Illness Score (Spearman’s rho: 0.723, p <. 001) | Cronbachs alpha: 0.93 Split-half Spearman-Brown coefficient: 0.91 Test-retest correlation coefficient: 0.83 | - |
|  |  | Santos et al, 2020 | - | Confirmatory factor analysis: Goodness of fit index (GFI) < 0.80; root mean square error approximation (RMSEA) ≤ 0.08; adjusted goodness of fit index (AGIF) ≤ 0.90; and chi-square measure < 0.05.27. The factorial load for each item ranged between 0.25 and 0.88. Factorial loads of factors 1 to 4 ranged from 0.43 to 0.96.  Convergent validity; moderate positive correlation of factors 2 (r= 0.512) and 3 (r= 0.407) to the Janis and Fields self esteem scale. Factor 1: r= 0.216 and factor 4: r=0.289. | Crohnbachs alpha: 0.85, for each factor range 0.59-0.67  Test-retest reliability: ICC: 0.903 | - |
|  | OCS | Kluka et al, 1996 | The OCS was developed based on reported concerns of stoma patients and input from 15 enterostomal therapy nurses. The OCS was pilot tested in four patients and two spouses of patients. | - | Cronbachs alpha: 0.92. Test-retest reliability (Spearman-s correlation): 0.73 | - |
|  | EAOE | Sousa et al, 2015 | The scale was developed based on a nursing approach and addressed 6 domains. Initial version underwent content validation by 25 experts and was pilot tested in 10 patients and pre-tested in 20 patients. | Factor analysis yielded 6 factors Pearsons correlation was significant between the total EAOE and most subscales. | Cronbachs alpha: 0.87, for each factor range 0.61-0.85. | - |
|  | ADM | Nordström et al, 1995 | - | Convergent validity: A close correlation between ADM and Sense of Coherence questionnaire was obtained (r=-0.54, p<0.01). Known-groups validity: there was a significant difference berween total ADM scores and type of ostomy (conventional/continent). | Cronbachs alpha: 0.96, item to total scale correlation range 0.17-0.88 | - |
| HRQoL in stoma patients | SQOLS | Baxter et al, 2006 | Content experts generated initial questions for the SQOLS. The SQOLS was pilot tested in a focus group with stoma patients. | Known-group-validity: 40 % believed that their stoma improved their QOL, 40 % believed that it worsened their QOL. Convergent validity: Significant correlations between the SQOLS and Physical Health Composite Scale of the SF-12 (0.54, p<0.0001) and Mental Health Composite Scale SF-12 (0.75, p<0.0001). | Cronbachs alpha: 0.89, for each domain range 0.76-0.89 Test-retest reliability: Intraclass correlation coefficient: 0.93, for each domain range 0.75-0.94 | - |
|  | COH-QOL-OQ | Anaraki et al,  2014 | Face validity was examined by two gastroenterologists and two psychologists. | Known-group validity: significant differences in social and spiritual well-being between cancer and non-cancer patients. | Cronbach alpha for all subscales range 0.74-0.85 | - |
|  |  | Gao et al,  2013 | Content validity was assessed by 10 experts with Item-Conten validity index for all items ranging 0.7-1.0. | A 4-factor confirmatory factor analysis fit the data well 𝛘2/df=1833, comparative fit index = 0.939, root mean square error of approximation = 0.048, nonnormed fit index =0.931I, incremental fit index = 0.940 | Cronbachs alpha: 0.931, for each domain range 0.708-0.898 Test retest reliability was >0.8 for all items. | - |
|  |  | Grant et al, 2004 | Face validity was established by an expert panel and a group of outside reviewer. | Exploratory factor analysis yielded 6 factors. Convergent validity: All factors were significantly correlated to single QOL item (range 0.24-0.76) Known-group-validity: COH-QOL-OQ total score and most subcales were significantly correlated to sexual concerns, emotional concerns, social support, work status and marrital status. | Cronbachs alpha: 0.95, the six factors ranging from 0.77 to 0.90. | - |
|  |  | Konjevoda et al,  2020 | - | A 4-factor confirmatory factor analysis fit the data well 𝛘2/df=1144.28,p<0.01 comparative fit index = 0.869, root mean square error of approximation = 0.077, nonnormed fit index =0.855 | Cronbachs alpha: 0.95, for each domain range 0.73-0.89 Test-retest reliability: 0.99 for total scale and all subscales | - |
|  |  | Krouse et al, 2006 | - | - | Cronbachs alpha: 0.96, for each domain range 0.8-0.94 | - |
|  |  | Mayadevi et al, 2019 | Face and content validity were evaluated by ten experts: two stoma nurses and eight oncologists and found adequate. | - | Cronbachs alpha: 0.96, for each domain range 0.85-0.91. Test-retest reliability: Intraclass correlation coefficient: 0.34, for each domain range 0.34-0.55 | - |
|  |  | Mohler et al, 2008 | - | Convergent validity: The physical, psychological and social well-being subscales were significantly correlated to the corresponding scales of the SF36-v2. Pearson correlations of 0.34, 0.71 and 0.43 respectively. | Cronbachs alpha: 0.94, for each domain range 0.8-0.9 | - |
|  |  | Santos et al,  2021 | - | Confirmatory factor analysis was performed. The first model, with 43 items, were not statistically significant for the model tested, with low factor loads of 0.06 and 0.05, respectively. Another confirmatory factor analysis was performed that excluded two items. This new model, with 41 items, presented acceptable adjustment indexes similar to the original model previously tested with parameters of χ2=0.0001, RMSEA = 0.08, AGFI = 0.62, and GFI = 0.66.  Convergent validity: The Psychological Well-Being domain of the COH-QOL-OQ had strong and statistically significant correlation coefficients of 0.64 (P= .000), 0.77 (P= .000), and 0.73 (P= .000), with the Physical domain, the Psychological domain, and the General QOL of the WHOQOL-Bref, respectively. With regard to the Physical Well-Being domain, the score resulted in a strong correlation between the Physical domain of the WHOQOL-Bref (r= 0.60; P< .001) and a moderate correlation with the General QOL of the WHOQOL-Bref(r= 0.53; P< .001). The Social well-being domain of the COH-QOL-OQ displayed a moderate correlation with the Social(r= 0.53; P< .001) and Environmental (r= 0.42; P< .001) domains and a strong correlation with the General QOL of the WHOQOL-Bref. | The internal consistency ( a measure of reliability) of the items was high with a Cronbach α of 0.92 for the total score and values ranging from 0.79 to 0.86 for the various domains of the COH-QOL-OQ. | - |
|  | CRC_QOL | Harisi et al, 2004 | The CRC_QOL was developed as a collaboration of physicians, surgeons, oncologists, psychologists, and the patients. A pilot test was performed in 100 patients. | Known-group-validity: Could discriminate between patients operated for cancer (ostomates and non-ostomates) and healthy population. | Cronbachs alpha for all subscales range 0.281-0.940 Test-retest reliability (Pearson correlation) for all questions ranging from 0.991-1.00 (p<0.01) | - |
|  | Stoma-QOL | Lai et al,  2018 | - | Fit statistics indicated poor fit of the unidimensional RaschGRS model (RMSEA: 0.124, TLI: 0.869, and CFI: 0.837). The chi-squared p-value less than 0.001 rejects the null hypothesis that the data and model fit perfectly. | Cronbachs alpha: 0.93 Item to scale correlation range 0.51-0.77 | - |
|  |  | Oliveira et al, 2017 | - | Convergent validity: A moderate and significant association was found between the Stoma-QOL and the SF-12. | Cronbachs alpha: 0.87 Test-retest reliability: Intraclass correlation coefficient: 0.85 | - |
|  |  | Prieto et al, 2005 | Potential items were formulated in English on the basis of the results of a series of semi-structured interviews with 169 adult stoma patients. A pilot test was performed in 182 patients. | Rasch analysis was performed on the categories used as response choices, indicating the 'distance' that separates the four response choices. These weights ranged from -0.94 to 1.66. Item parameters by country also fitted to the Rasch model(Infit MNSQ<1.3) and had very similar item calibrations: ICC of the item calibrations by country was 0.81 (0.67–0.91 95% CI). The classical item discrimination index for the 20 items of the questionnaire ranged from 0.51 to 0.67. The mean response choice for each item (difficulty index) ranged from 2.11 to 3.60, which suggests that all items are moderately spread around the centre of the four response choices (1.Always; 2.Sometimes; 3.Rarely; 4.Not at all) | Cronbachs alpha: 0.92 Test-retest reliability (Pearsons correlation) over 0.88 (p<0.01). | - |
|  |  | Canova et al, 2013 | - | Rasch analysis of the 251 patients supported the Stoma Care QoL scale questionnaire as a global measure of QoL in stoma patients. The model showed acceptable goodness-of-fit (log-likelihood chi-square testP-value<0.01). The items’ estimates,or their ‘weights’, varied between1.2 to 0.85. | Cronbachs alpha: 0.9, above 0.8 for all subgroups. | - |
|  | The Ostomy-Q | Nafees et al,  2017 | The Ostomy-Q was developed based on a litterature review and existing tools. It was pilot tested in 5 stoma-patients. After revision content validity was assessed with the same 5 patients and 2 new users. | Factor analysis yielded 6 factors. Convergent validity: All domains of the Ostomy-Q had a positive association with the 2 domains of OAI-23 and the total score of the Ostomy-Q (P <.01) in the sample. All domain and total scores on the Ostomy-Q exceeded the correlation benchmark of 0.40 against the OAI-23 (and subscales). The total score of the Ostomy-Q and the OAI-23 had the highest correlation >0.75 (P <.01), and all of the domains of the Ostomy-Q had significant correlations with the original Stoma-QoL and ex-ceeded the criterion (P <.01). | Cronbachs alpha: 0.889, for each domain range 0.685-0.781. Test-retest reliability: Intraclass correlation coefficient: 0.909, for each domain range 0.741-0.830. | Sensitivity and MID estimates. The effect sizes varied from 0.35 to 0.70, suggesting moderate to high effect sizes for each domain. The estimates of standardized response means were in alignment. |
| Self efficacy in stoma patients | SSES | Bekkers et al, 1996 | Development: Selection of relevant items was based on Bandura's theory of self-efficacy, other self-efficacy measures for chronically ill patients, existing psychosocial data on stoma patients and clinical experience of enterostomal therapists and medical specialists. | Factor analysis yielded 2 factors with 61 % explained variance.  Convergent validity: The intercorrelation to the Psychosocial Adjustment to Ilness Scale is relatively high (Pearson r = .73). | Cronbachs alpha: 0.94 and 0.95 for the two subscales. | - |
|  |  | Karaçay et al,  2020 | According to expert opinions, the CVI of the Turkish version of thescale was .96, indicating excellent content validity | To determine construct validity exploratory factor analysis (EFA) and confirmatory factor analysis (CFA) were carried out. Model fit of item–factor relationship obtained by EFA was tested by CFA. Thus, it was determined that the factor structure of the origi- nal Stoma SE Scale was confirmed in the Turkish sample. In the CFA, the fit index of the Turkish form's two-factor model was examined. It was found that scale's fit indices were significant after modification ( χ2 = 485.887; df = 202 ,P=.001;P<.01) The fit index values were found to be NC = 2.405; GFI = 0.904; RMSE = 0.090; CFI = 0.903; NFI = 0.846; RFI = 0.824; and IFI = 0.904. | Cronbachs alpha: 0.95, for each domain 0.92 and 0.93. Test-retest reliability: Item correlation coefficient 0.97, for each domain 0.97 | - |
|  | CC-OSCI | Villa et al, 2019 | Developing the CC-OSCI Villa et al followed four steps in accordance with adetailed literature review of self-care in ostomy patients and theircaregivers, Riegel's theory of self-care and self-care in ostomypatients. In the first two steps, we defined the construct of self-careand created the tools, respectively. In the third step, we defined andselected the pertinent indicators; in the last step we performed aback-translation into English. The CVI was 93 % for the CC-OSCI. | Factor analysis yielded 3 factors. Convergent validity: Each scale of the CC-OSCI was correlated moderately and statistically significant with the Stoma-QoL, except for the confidence scale. The Mann-Whitney-U-test was used for these calculations. | Cronbachs alpha: 0.972, for each domain range 0.912-0.972 | - |
|  | OSCI | Villa et al,  2019 | Developing the OSCI Villa et al followed four steps in accordance with adetailed literature review of self-care in ostomy patients and theircaregivers, Riegel's theory of self-care and self-care in ostomypatients. In the first two steps, we defined the construct of self-careand created the tools, respectively. In the third step, we defined andselected the pertinent indicators; in the last step we performed aback-translation into English. The CVI was 95 % for the OSCI. | Factor analysis yielded 3 factors. Convergent validity: Each scale of the OSCI was correlated moderately and statistically significant with the Stoma-QoL, except for the confidence scale. The Mann-Whitney-U-test was used for these calculations. | Cronbachs alpha: 0.975, for each domain range 0.930-0.965 | - |
| Stoma acceptance | ACHC (Stoma) | Lim et al,  2017 | The CVI was 0.94, demonstrating the validity content of the SC-ACHC (Stoma) scale. | Convergent validity: ACHC (stoma) was posetively correlated to the Chinese version of the EQ-5D-5L (European QOL-5 dimensions-5 levels) utility score (r=0.63, p<0.01) and general visual analogue scale (r=0.7, p<0.01) | Cronbachs alpha: 0.846 Test-retest reliability: Item correlation coefficient 0.997 | - |
|  | SAQ | Bagnasco et al, 2017 | The SAQ was developed after 10 focus groups with a total of 350 stoma care nurses, and pilot tested in a 104 patients. | Mokken scaling: H=0.47, Rho=0.88, HT=0.41 | Internal consistency: Since the lower-bound level of scalability is set at 0.3, the confidence interval for items should contain 0.3, whereas for item pairs, the confidence intervals should not contain 0. There was found to be insufficient evi-dence to support the scalability of items 6 and 16, and that of item pairs 4, 6, and 14 with item 16. | - |
|  | CDS | Jin et al,  2020 | Development and face validity: Litterature review and focus group interviews followed by multiple rounds of expert panel assessment and research team modification until consensus. The CVI was used to examine content validity of each item (I-CVI) and scale (S-CVI). CVIs<0.78 was removed. | Exploratory factor analyses yielded 2 factors.  Confirmative factor analyses: (χ^2^=226.284, df=208, p<.001), comparative fit index=0.99, Tucker-Lewis indeax=0.99 and RMSEA=0.02  Convergent validity: Item factor loadings between 0.65-0.81, average variance extracted for subscales 0.55 and 0.54. | Cronbachs alpha: 0.94 and 0.91 for subscales.  Composite reliability 0.94 and 0.92 for subscales. | - |
| Stoma function | CIS | Thyø et al,  2016 | The CIS was developed based on a litterature review, discussion and revisions by a panel of experts and two pilot tests. | Convergent validity: We found statistically significant differences in EORTC-QLQ for all functional and symptom scales comparing the Minor CI group and the Major CI group. The CIS was tested against an overall QOL anchor question yielding a sensitivity and specificity of 85,7 % and 59,5 % respectively (AUC 0.7964). | - | - |
|  |  | Kristensen et al, 2020 | - | Convergent validity: Pearson correlation coefficient of0.41 between the CI score and Physical Component Summary and a Pearson correlation coefficient of0.39 between the CI score and Mental Component Summary, and for each of the patient subgroups. | - | - |
|  |  | Kristensen et al, 2021 | - | Convergent validity: The differences in scale scores for the EORTC QLQ-C30 between the CI groups were all clinically relevant. ROC analyses: Sensitivity ranged between 42% and 78% for the anchor question concerning adaptation, between 66% and 82% for the anchor question concerning embarrassment and between 60% and 89% for the anchor question concerning restrictions in daily activities.  Discriminative validity: For groups showing a difference in the rate of patients reporting impaired HRQoL, the CI score correspondingly showed significantly higher CI scores in the groups with inferior HRQoL except for age groups where the difference in CI score did not reach significance (p = 0.0974). Similarly, for subgroups that reported ‘some/a lot’ impact on HRQoL at equal rates no difference in CI scores were present, with the exception of patients with Clavien–Dindo ≥II complications who had significantly higher CI scores but did not report an impact on HRQoL more often. | Test-retest reliability: ICC scores showed moderate reliability in Sweden and the Netherlands for both sum-score (0.663 and 0.701, respectively) and item-level scores (0.640 and 0.749,respectively) and for sum-score in Denmark (0.705). Reliability was good on item level in Denmark (0.783) and excellent regarding both sum-score (0.919) and on item level (0.898) in Spain. |  |
|  | CCFOFI | Colquhoun et al, 2006 | - | Convergent validity: Overall, the CCFOFI correlated with all eight scales of the SF-36 as well as the PCS andMCS, with all variables demonstrating values of P<0.003. | - | - |
|  | BFI | Wendel et al,  2014 | - | In factor analysis of the ostomy group, Urgency/Soilage, Frequency, and Dietary explained 24%, 19%, and 27% of variance, respectively. Convergent validity: BFI showed significant correlations with both COH-QOL-OQ and SF-12v2 as well as most subscales. | Cronbach’s alphas: In the ostomy group: Urgency/Soilage (alpha = 0.74) and Dietary (alpha = 0.83) were found to be internally consistent, but Frequency (alpha = 0.50) was not. | - |
|  | PSAT | Sodhi et al,  2012 | PSAT was formed. It consisted of 6 parameters such as colour, tissue type, type of exudate, bleeding, wound edges and hydration.  Content validation was performed using a modified Delphi’s approach.The CVI of tool was 0.8. | - | - | - |
|  | OLIT | Nafees et al, 2018 | The OLIT was developed based an a litterature review and input from 41 patients and 6 experts. Content validity with 20 patients. | Convergent validity: The OLIT subscales was significantly correlated to SF36 subscales, Ostomy-Q and OAI-23 for all hypothesized relationships (Pearsons correlation) range 0.442-0.771. | Cronbachs alpha for all subscales range 0.77-0.93. Test-retest reliability: Item correlation coefficients for all subscales 0.582-0.885. | - |
| ACHC (Stoma): Acceptance of Chronic Health Conditions Stoma Scale; ADM: Acceptance of Disability Scale Modified; BFI: Bowel Function Index (modified); CC-OSCI: Caregiver Contribution to Self-Care in Ostomy Patient Index; CCFOFI: Cleveland Clinic Florida Ostomy Function Index; CDS: Colostomy Disgust Scale; CIS: Colostomy Impact Score; COH-QOL-OQ: City of Hope Quality of Life Ostomy Questionnaire; CRC_QOL: Colorectal cancer specific questionnaire on quality of life; EAOE: Elimination Ostomy Adjustment Scale; OAI-23: Ostomy Adjustment Inventory-23; OAS: Ostomy Adjustment Scale; OCS: Ostomy Concerns Scale; OLIT: Ostomy Leak Impact Tool; OSCI: Ostomy Self-Care Index; PSAT: Peristomal Skin Assessment Tool; SAQ: Stoma Acceptance Questionnaire; SQOLS: Stoma Quality Of Life Scale; SSES: Stoma Self-Efficacy Scale; Stoma-QOL: Stoma Quality of Life Questionnaire. | | | | | | |
