# Supplementary material for: Patient reported outcome measures assessing quality of life in patients with an intestinal stoma: A systematic review
Source: Colorectal Dis. 2022 Jun 16;24(10):1128–39. doi: 10.1111/codi.16202 (PMC9796962; doi:10.1111/codi.16202)
Supplement: Supplementary file 3 — Data S1 [file CODI-24-1128-s002.docx]

# Search strategy

## MedLine

|  |  | Search terms | Block definition |
| --- | --- | --- | --- |
|  | #1 | Stoma OR stomas OR ostomy OR ostomies OR ostomate OR "ileal conduit" OR enterostomy OR enterostomies OR colostomy OR colostomies OR ileostomy OR ileostomies OR jejunostomy OR jejunostomies | Stoma |
|  | #2 | "Ostomy"[Mesh] OR "Surgical Stomas"[Mesh] |  |
|  | #3 | #1 OR #2 |  |
|  | #4 | validation OR validity OR reliability OR “psychometric properties” | Validation |
|  | #5 | measure* OR scale OR scales OR score OR scores OR assessment OR survey OR surveys OR questionnaire OR questionnaires OR tool OR tools "patient reported outcome" | PROM |
|  | #6 | "Validation Studies as Topic"[Mesh] OR "Reproducibility of Results"[Mesh] OR "Weights and Measures"[Mesh] OR "Surveys and Questionnaires"[Mesh] |  |
|  | #7 | #5 OR #6 |  |
|  | #8 | #3 AND #4 AND #7 |  |

### Compact search string

((((((("Ostomy"[Mesh] OR "Surgical Stomas"[Mesh]))) OR ((Stoma OR ostomy OR ostomies OR ostomate OR "ileal conduit" OR enterostomy OR enterostomies OR colostomy OR colostomies OR ileostomy OR ileostomies OR jejunostomy OR jejunostomies)))) AND ((validation OR validity OR reliability OR “psychometric properties”)) OR (("Validation Studies as Topic"[Mesh] OR "Reproducibility of Results"[Mesh]))) AND ((((measure* OR scale OR scales OR score OR scores OR assessment OR survey OR surveys OR questionnaire OR questionnaires OR tool OR tools OR "patient reported outcome"))) OR (("Weights and Measures"[Mesh] OR "Surveys and Questionnaires"[Mesh]))))

## Embase

|  |  | Search terms | Block definition |
| --- | --- | --- | --- |
|  | #1 | Stoma OR stomas OR ostomy OR ostomies OR ostomate OR "ileal conduit" OR enterostomy OR enterostomies OR colostomy OR colostomies OR ileostomy OR ileostomies OR jejunostomy OR jejunostomies | Stoma |
|  | #2 | "Ostomy"/exp OR stoma/exp |  |
|  | #3 | #1 OR #2 |  |
|  | #4 | validation OR validity OR reliability OR “psychometric properties” | Validation |
|  | #5 | measure* OR scale OR scales OR score OR scores OR assessment OR survey OR surveys OR questionnaire OR questionnaires OR tool OR tools OR "patient reported outcome" | PROM |
|  | #6 | 'validation study'/exp OR 'reproducibility'/exp OR 'standard'/exp OR 'questionnaire'/exp |  |
|  | #7 | #5 OR #6 |  |
|  | #8 | #3 AND #4 AND #7 |  |

### Compact search string

(('ostomy'/exp OR 'stoma'/exp) OR (stoma OR stomas OR ostomy OR ostomies OR ostomate OR 'ileal conduit' OR enterostomy OR enterostomies OR colostomy OR colostomies OR ileostomy OR ileostomies OR jejunostomy OR jejunostomies)) AND (validation OR validity OR reliability OR 'psychometric properties') AND ((measure* OR scale OR scales OR score OR scores OR assessment OR survey OR surveys OR questionnaire OR questionnaires OR tool OR tools OR 'patient reported outcome') OR ('validation study'/exp OR 'reproducibility'/exp OR 'standard'/exp OR 'questionnaire'/exp))

## CINAHL

|  |  | Search terms | Block definition |
| --- | --- | --- | --- |
|  | #1 | Stoma OR stomas OR ostomy OR ostomies OR ostomate OR "ileal conduit" OR enterostomy OR enterostomies OR colostomy OR colostomies OR ileostomy OR ileostomies OR jejunostomy OR jejunostomies | Stoma |
|  | #2 | (MH "Ostomy+") |  |
|  | #3 | #1 OR #2 |  |
|  | #4 | validation OR validity OR reliability OR “psychometric properties” | Validation |
|  | #5 | (measure* OR scale OR scales OR score OR scores OR assessment OR survey OR surveys OR questionnaire OR questionnaires OR tool OR tools OR "patient reported outcome") | PROM |
|  | #6 | (MH "Validation Studies") OR (MH "Reproducibility of Results") OR (MH "Weights and Measures+") OR (MH "Surveys+") |  |
|  | #7 | #5 OR #6 |  |
|  | #8 | #3 AND #4 AND #7 |  |

### Compact search string

((Stoma OR stomas OR ostomy OR ostomies OR ostomate OR "ileal conduit" OR enterostomy OR enterostomies OR colostomy OR colostomies OR ileostomy OR ileostomies OR jejunostomy OR jejunostomies) OR (MH "Ostomy+")) AND (validation OR validity OR reliability OR “psychometric properties”) AND ((measure* OR scale OR scales OR score OR scores OR assessment OR survey OR surveys OR questionnaire OR questionnaires OR tool OR tools OR "patient reported outcome") OR (MH "Validation Studies") OR (MH "Reproducibility of Results") OR (MH "Weights and Measures+") OR (MH "Surveys+"))

## COCHRANE

|  |  | Search terms | Block definition |
| --- | --- | --- | --- |
|  | #1 | Stoma OR stomas OR ostomy OR ostomies OR ostomate OR "ileal conduit" OR enterostomy OR enterostomies OR colostomy OR colostomies OR ileostomy OR ileostomies OR jejunostomy OR jejunostomies | Stoma |
|  | #2 | ‘Ostomy’ [Mesh] |  |
|  | #3 | ‘Surgical Stomas’ [Mesh] |  |
|  | #4 | #1 OR #2 OR #3 |  |
|  | #5 | validation OR validity OR reliability OR “psychometric properties” | Validation |
|  | #6 | measure* OR scale OR scales OR score OR scores OR assessment OR survey OR surveys OR questionnaire OR questionnaires OR tool OR tools "patient reported outcome" | PROM |
|  | #7 | ‘Validation Studies as Topic’ [Mesh] |  |
|  | #8 | ‘Reproducibility of Results’ [Mesh] |  |
|  | #9 | ‘Weights and Measures’ [Mesh] |  |
|  | #10 | ‘Surveys and Questionnaires’ [Mesh] |  |
|  | #11 | #6 OR #7 OR #8 OR #9 OR #10 |  |
|  | #12 | #4 AND #5 AND #11 |  |
